# Asthma-related mortality in Italian children and adolescents

**DOI:** 10.1186/s13052-026-02303-9

**Published:** 2026-08-05

**Authors:** Francesca Saretta, Mattia Giovannini, Angela Klain, Luca Pecoraro, Stefania Arasi, Simona Barni, Lucia Caminiti, Riccardo Castagnoli, Mariannita Gelsomino, Lucia Liotti, Carla Mastrorilli, Francesca Mori, Gianluigi Marseglia, Elio Novembre

**Affiliations:** 1 Pediatric Department, Azienda Sanitaria Universitaria Friuli Centrale, 33100 Udine, Italy; 2 Allergy Unit, Meyer Children’s Hospital IRCCS, 50139 Florence, Italy; 3 Department of Health Sciences, University of Florence, 50139 Florence, Italy; 4 Department of Woman, Child and General and Specialized Surgery, University of Campania Luigi Vanvitelli, 80138 Naples, Italy; 5 Pediatric Unit, Hospital “Vito Fazzi”, 73100 Lecce, Italy; 6 Pediatric Allergology Unit, Bambino Gesù Children’s Hospital (IRCCS), 00165 Rome, Italy; 7 Department of Human Pathology in Adult and Development Age “Gaetano Barresi”, Allergy Unit, Department of Pediatrics, AOU Policlinico Gaetano Martino, 98124 Messina, Italy; 8 Department of Clinical, Surgical, Diagnostic and Pediatric Sciences, University of Pavia, 27100 Pavia, Italy; 9 Pediatric Clinic, San Matteo IRCCS Polyclinic Foundation, 27100 Pavia, Italy; 10 Department of Life Sciences and Public Health, Pediatric Allergy Unit, University Foundation Policlinico Gemelli IRCCS, Catholic University of the Sacred Heart, 00168 Rome, Italy; 11 Pediatric Unit, Department of Mother and Child Health, Salesi Children’s Hospital, 60123 Ancona, Italy; 12 Pediatric Hospital Giovanni XXIII, Pediatric and Emergency Department, AOU Policlinic of Bari, 70126 Bari, Italy

**Keywords:** Asthma, Mortality, Children, Adolescents, Pediatrics, Italy

## Abstract

Despite the progress made in the last few decades in diagnosis and therapy, children and adolescents still die of asthma worldwide. In this study, we collected all asthma-related death events in Italian children and adolescents (0–19 years old) from 2013 to 2023 through the Italian National Statistical Database. 46 deaths were recorded, with a mortality rate of 0.39 per million individuals aged 0–19 years old (population denominator: 10.7 million), with the highest number of deaths observed in North-East Regions (36.9%) and 39/46 (84.7%) deaths between 10 and 19 years old. Factors associated with fatal asthma are also discussed. Targeted preventive strategies and further research are called for, particularly in high-incidence regions and adolescents.

## Introduction

Despite the continuous progress made in the last few decades in the diagnosis, management, and therapy of asthma, children and adolescents still die of this chronic respiratory disease.

The rate of mortality due to asthma varies greatly among different Countries. Data regarding death due to asthma in pediatric patients are scattered because no official worldwide registry exists. As reported by the European Union Statistics Database [1], in Italy a 0.6 per 100,000 individuals mortality rate per year was reported both for males and females (0.5 for males and 0.7 for female, respectively); however, these data included all age ranges.

Several risk factors have been identified in the literature as being associated with increased risk of asthma-related mortality: previous severe exacerbations requiring hospitalization or intensive care or mechanical ventilation, poor adherence to therapy, over reliability on short-acting beta-2 agonists (SABA), low socio-economic status and male sex [2–5]. Moreover, environmental factors, such as air pollution and allergen exposure have been implicated [6].

The primary aim of the study was to collect and analyze all asthma-related deaths in children and adolescents (0–19 years old) from 2013 to 2023 through the ISTAT (Istituto Nazionale di Statistica) Italian National Institute of Statistics, with the goal of advocating preventive strategies and public health intervention on this topic.

## Methods

We performed a retrospective search through the ISTAT database, collecting all deaths attributed to asthma that occurred in children and adolescents (0–19 years) from January 1st 2013 to Decembrer 31st 2023 in Italy. Data extractions were last performed on February 19th 2026.

All deaths coded with an ICD-10 asthma-related code were considered: J45.0 predominantly allergic asthma, J45.1 nonallergic asthma, J45.8 mixed asthma, J45.9 unspecified asthma, and J46 status asthmaticus. We included all these codes to record the full range of asthma-related mortality; however, we are aware that J45.9 may include cases of uncertain diagnosis, and J46 specifically represents acute life-threatening asthma attack, but given the small number of these cases, separated statistical analysis was not accurate and not provided.

Owing to the age classification set in the ISTAT database, all individuals up to 19 years of age were considered. It should be noted that this age range includes late adolescents who may legally be considered adults; however, for consistency with ISTAT database age classification, we maintained 0–19 years age range.

All death events were analyzed according to the following categories:

1. *Macroregion* as reported in the ISTAT Database: North-West Regions (Piedmont, Lombardy, Aosta Valley, Liguria), North-East Regions (Veneto, Friuli-Venezia Giulia, Trentino-Alto Adige, Emilia-Romagna), Central Regions (Tuscany, Marche, Lazio, Umbria), South Regions (Abruzzo, Molise, Campania, Basilicata, Puglia, Calabria), Islands (Sicily, Sardinia)
2. Age *range*: 0 years (<12 months), 1-4 years, 5-9 years, 10-14 years, 15-19 years
3. Sex: female and male

Mortality rates (*crude death rate*) were expressed as deaths per million individuals aged 0–19 years old. Population denominators were obtained from ISTAT database using year-specific resident population (aged 0–19 years old) figures for each corresponding year of the study period. Descriptive statistics (counts and percentages) were used; no inferential testing was performed, given the small number of events. It is important to note that death certificate coding have some limitations since deaths occurring outside hospital setting or without testimony may be coded as “cardiac arrest” or “respiratory failure” or “unknown cause” rather than “asthma”, potentially leading to underestimation of true asthma mortality. Additionally, ISTAT coding typically reports the primary probable cause of death, although some deaths may include asthma as contributory rather than the main cause.

These data are anonymized and publicly available; therefore, formal ethical approval was not required.

## Results

**Fig. 1:**
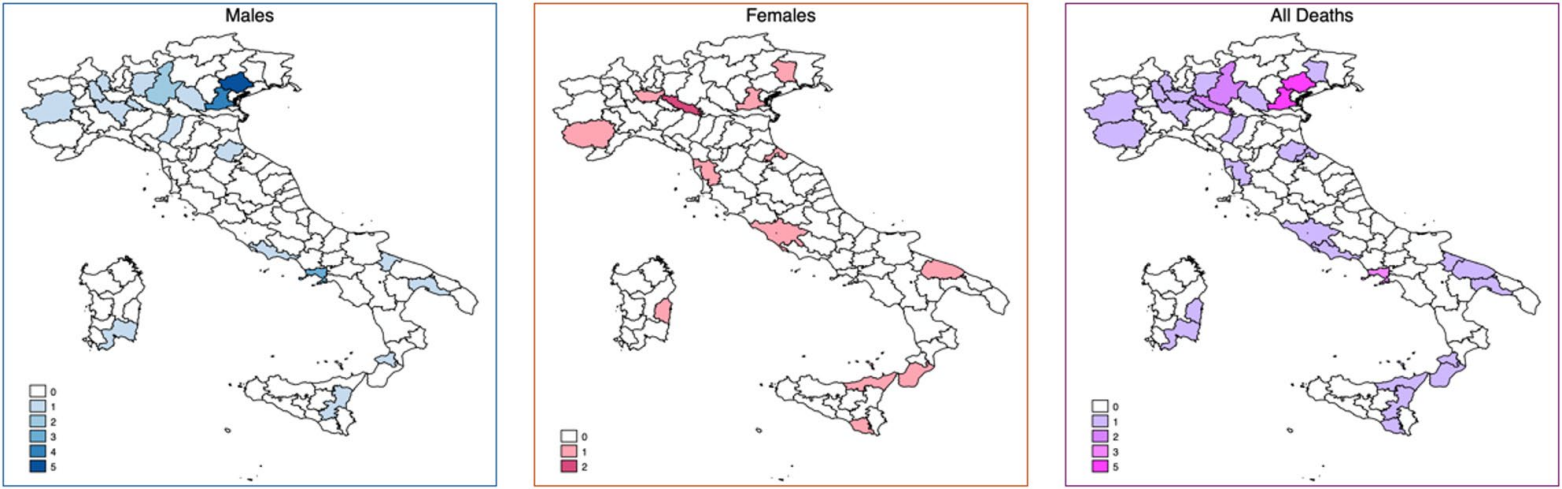
Number of asthma-related deaths by sex and age group in Italian children and adolescents (0–19 years) from 2013 to 2023. Bars represent the number of male deaths (blue), female deaths (red), and total deaths (purple). Data shown up to 2022; 2023 data is not plotted separately due to incomplete year coverage but included in the total analysis

From 01/01/2013 to 31/12/2023 (11 years), 46 asthma-related deaths in children and adolescents (0–19 years) were recorded in Italy. The distribution by age group and gender is presented in Fig. 1 and Table 1.

**Table 1:** Asthma-related deaths in pediatric age (0–19 years) from 2013 to 2023 in Italy by age groups and gender

Age group	Deaths (*n*)	% of total	Female %	Male %
< 1 y	0	0%	0%	0%
1–4 y	5	10.9%	20%	80%
5–9 y	2	4.4%	50%	50%
10–14 y	17	36.9%	47%	53%
15–19 y	22	47.8%	32%	68%
**Total**	**46**	**100%**	**37%**	**63%**

Almost two-thirds (30/46, 65,2%) of deaths occurred in Northern Italy (13/46 in North-West Regions and 17/46 in North-East Regions). The remaining deaths occurred in the Central Regions (3/46, 6.5%), %) in the Southern Regions (8/46, 17.4%), and in the Islands (5/46, 10.9%). The distribution by macroregion and respective mortality rates is shown in Table 2.

**Table 2:** Asthma-related deaths in pediatric age (0–19 years) from 2013 to 2023 in Italy by macroregion and respective mortality rates

Macroregion	Deaths (*n*)	% of total	Mortality Rate/million/individuals 0–19 years
North-East	17	36.9%	0.75
North-West	13	28.3%	0.42
Centre	3	6.5%	0.13
South	8	17.4%	0.28
Islands	5	10.9%	0.38
**Italy total**	**46**	**100%**	**0.39**

Regarding sex, in all Italy, 17/46 deaths (37%) were females and 29/46 (63%) were males. Among females, the highest number of deaths was observed between 10 and 14 years old (8/17 deaths, 47%), followed by between 15 and 19 years old (7/17 deaths, 41.2%). Among males, the highest number was observed between 15 and 19 years old (15/29 deaths, 51.7%), followed by 10–14 years old (9/29 deaths, 31%). The predominance of deaths in adolescents (10–19 years) and males aligns with international data, where older children and boys experience higher fatality rates [7]. This age group is the one in which adherence to therapy wanes and self-managed rescue medication overuse becomes common, both established factors associated in the literature [8] with poor asthma control. Due to the small number of events, we are aware that in our study calculated mortality rates for specific regions, age and sex groups could be only indicative; therefore, these findings should be interpreted with caution.

Regarding temporal trends, from 2003 to 2012, in the ISTAT database 38 asthma-related deaths were recorded (10 girls, 29 boys; data not shown in the Results but available through the database), while from 2013 to 2022, 43 events were recorded (13 girls, 29 boys). In 2023 only 3 deaths have been reported. The modest increase in the last decade may reflect improved reporting rather than a true rise in mortality; statistical significance cannot be assessed due to the limited numbers, and we do not draw any conclusions regarding temporal changes in mortality.

For comparative purposes with other Countries, we have summarized in Table 3 a few international pediatric asthma mortality rates as reported in recent literature.

**Table 3:** Mortality rates for asthma that occurred in pediatric age (0–19 years) in some national reports

Nation	Deaths (*n*)	Years ranges	Children 0–19 years old (*n* per year)	Mortality Rate death/year/million individuals aged 0–19 years old
USA [9]	155	annually	84.5 million	1.83
England [10]	54	2019–2023	12.4 million	1
Ireland [11]	11	2006–2016	1.3 million	0.84
Finland [12]	4	1999–2015	1.2 million	0.55
**Present study**	**46**	**2013–2023**	**10.7 million**	**0.39**

A brief analysis of ICD-10 code distribution revealed that cases coded as J46 (status asthmaticus) represented a small subset of the total deaths but due to the very small number of events in this subgroup, we conclude that a separate statistical analysis would not be significant enough to infer any further conclusions. However, we recognize that these cases likely represent more acute, life-threatening asthma events compared to deaths attributable to chronic asthma.

## Discussion

This nationwide analysis reveals that asthma-related mortality in Italian children and adolescents occurs at a rate of 0.39 deaths per million individuals aged 0–19 years, which is lower than rates reported in other Countries (see Table 3). Most fatal cases were registered in adolescents aged 10–19 years (84.7%) and in males (63%), patterns consistent with international literature.

The identification of risk factors associated with fatal asthma in childhood has been proposed in multiple studies [2–5].

1. History of near-fatal asthma requiring intubation and mechanical ventilation
2. Insufficient or poor adherence to controller therapy or inadequate assessment
3. Severe asthma not currently treated with inhaled glucocorticoids
4. Overdependence on short-acting inhaled b2 agonists (SABA), especially those who use more than one canister of salbutamol (or equivalent) monthly
5. Socioeconomical issues and low sociodemographic index
6. Previous severe exacerbation requiring admission or intensive care
7. Previous anaphylactic reactions especially if associated with food allergy
8. Difficult-to-treat asthma
9. Male sex in childhood

Unfortunately, due to the restriction of ISTAT registry, whose available individual-level data are limited as their inclusion in rare events could pose the risk of identifying individuals and violating GDPR protections, our study could not evaluate these factors. It is, however, important to state that understanding these risk factors remain essential for asthma prevention and management, as stated by *Stephenson et al.* highlighted [13].

Finland had established a unified national asthma management program, which successfully reduced asthma mortality through early diagnosis, guided self-management, patient education, and scientific research promotion [14]. The absence in Italy of a similar coordinated national strategy, engaging not only research centers and hospitals but also family physicians and pediatricians, may contribute to outcome differences. The European Asthma Research and Innovation Partnership (EARIP) aims to decrease asthma mortality by 50% within 2035 through harmonization of asthma education [15] but Italy still lacks a nationwide integrated plan specifically addressing pediatric populations, although National Pediatric Medical Associations are actively working on it.

### Adolescents-specific risk factors

We observed a higher number of deaths in adolescents (10–19 years) which warrants dedicated attention. As already known in literature [7, 8] this age group is characterized by several risk factors: risk-taking behaviors and disease denial; stigma associated with asthma medication use; poor follow-up and therapeutic adherence; over-reliance on SABA without adequate controller therapies; mental health comorbidities such as anxiety and depression. Moreover, transition from pediatric to adult healthcare services could be heterogenous and scattered. These data reinforce the key concept that preventive interventions should be specifically targeted toward adolescents and young adults’ population, potentially involving school-based programs and enhanced family pediatricians support during the transition to adult care.

### Regional variations and potential contributing factors

We observed a geographic clustering, with North-East Regions showing the highest mortality rate (0.75 per million), however, we must underline that no environmental, socioeconomical or health care access data were available in the ISTAT database, due to privacy regulation and therefore, any explanation for regional differences remain, at this point, hypothetical.

Exposure to air pollutants, particularly ozone and PM 2.5, has been associated with near-fatal and fatal asthma in children, as reported by *Varghese et al.* [6]. In the National Child Mortality Database Thematic Report by *Stoianova et al.* [4] it is clearly stated that all British children who died of asthma have been exposed to air pollution above WHO guideline limits.

The distribution of immigrant populations may play a role since Northern Regions host a higher proportion of immigrants, and socioeconomic disparities, limited healthcare access and cultural barriers may affect healthcare and asthma outcomes in these communities [16, 17]. However, as mentioned above, ISTAT database does not provide information on ethnicities or countries of origin. Therefore, this hypothesis requires confirmation through future research with individual-level data linking mortality records to socioeconomic and demographic variables.

Lastly, although Italy has a national public healthcare system which relies on common coding guidelines, potential regional differences in individual physician diagnosis and coding practices could also had influence mortality estimations and should be considered when interpreting geographical variation.

## Strength and limitations of the study

This study had several strengths: it has nationwide coverage, uniform ICD-10 coding, and data are stratified by age, sex, and macroregion. It also has important limitations that must be acknowledged but these should be rather taken as encouragement for future research.

First, we relied solely on death certificate coding, which may result in underestimation of true asthma mortality since deaths occurring without testimony may be further coded as “cardiac arrest” or “respiratory arrest” or “unknown cause” rather than “asthma”. Second, misclassification could be particularly relevant for children under 5 years of age, an age group in which it is difficult to diagnose asthma with certainty since evidence-based asthma diagnosis is unreliable; attributing those deaths to “asthma” based on administrative coding may be incorrect in this age group. Third, the ISTAT database do not report personal data such as ethnicity, allergies, gestational age, underlying diseases that could worsen asthma control, environmental exposure (cigarette smoke, pollutants or indoor allergens), accessibility to emergency hospital services, adherence to treatments, family income. The unavailability of these data may misclassify the cause of death and limit the power of the statistical comparisons. Fourth, the very low absolute number of events means that mortality rates for specific regions and age groups are statistically weak and should be interpreted with caution. Finally, interpretation of temporal trends cannot be made with statistical confidence due to the small sample size. Despite these limitations, these remain the most comprehensive national data on asthma-related death in Italian children and adolescents and should represent a strong motivation for preventive strategies and future research. Similarly, it would be useful to involve the family pediatrician and the general practitioner in the diagnosis and management of all children and adolescents with moderate-severe forms of asthma, especially those with difficulty in following the therapy and with family compliance issues.

## Conclusions

Asthma-related mortality in Italy remains rare but shows a North-Eastern clustering and predominates in older children and males. The majority of deaths occurred in adolescents, highlighting the critical need for targeted preventive interventions in this age group and dedicated strategies, such as strengthening of asthma management, SABA overuse tracking, and systematic and facilitated transition-of-care in adolescents. We recommend the establishment of a nationwide pediatric asthma mortality registry with additional regional surveillance, including all medical personnel involved in children and adolescents’ healthcare. Future research should also aim to integrate environmental, socioeconomic, and clinical data to better understand contributing factors and develop evidence-based preventive policies, particularly in high-incidence regions.

## Data Availability

All datasets used for the current study available from the corresponding author on reasonable request.
